# Polysaccharide from Fuzi (FPS) Prevents Hypercholesterolemia in Rats

**DOI:** 10.1186/1476-511X-9-9

**Published:** 2010-01-28

**Authors:** Xiongqing Huang, Juan Tang, Qin Zhou, Hanping Lu, Yiling Wu, Weikang Wu

**Affiliations:** 1 Department of Anesthesiology, The First Affiliated Hospital, Sun Yat-sen University, Guangzhou 510080, PR China; 2 Guangzhou Critical Care Medicine Department, Guangzhou Women and Children's Medical Center, Guangzhou 510120, PR China; 3 Department of Nuclear Medicine, Zhongshan Medical College, Sun Yat-sen University, Guangzhou 510089, PR China; 4 Hebei Yiling Pharmaceutical Research Institute, Shijiazhuang 050035, PR China; 5 Institute of Integrated Traditional Chinese and Western Medicine, Zhongshan Medical College, Sun Yat-sen University, Guangzhou 510089, PR China

## Abstract

**Background and aim:**

Polysaccharide from fuzi (FPS), a Chinese herbal medicine extract, has been demonstrated to exert lipid lowering affects. In this study we examined potential mechanisms underlying this affect, specifically alterations in expression of the LDL-receptor (LDL-R), 3-hydroxy-3-methyl glutaryl (HMG)-CoA reductase and cytochrome P450 7α-1 (CYP7α-1), using a rat model of hypercholesterolemia.

**Methods and results:**

Male rats were fed either a normal or high cholesterol (HC) diet for two-weeks. Half of the rats on the HC diet were orally gavaged with FPS (224 mg/kg, 448 mg/kg or 896 mg/kg diet) daily. Serum lipid levels were quantified at end of the study period as were liver levels of LDL-R protein and mRNA expression of CYP7α-1 and HMG-CoA. Serum cholesterol and LDL-C concentrations were significantly elevated from control in HC rats, but not in those treated with FPS (P < 0.05). LDL-R expression was significantly decreased in the HC group compared to control (P < 0.05), but significantly increased in the FPS group (P < 0.05). HMG-CoA mRNA levels were significantly increased in the HC group compared both other groups (P < 0.05), while CYP7α-1 expression was significantly higher in the FPS group compared to both other groups (P < 0.05).

**Conclusion:**

These findings suggest that the cholesterol lowering effect of FPS in hypercholesteremic rats is caused at least in part by increased hepatic LDL-R and CYP7α-1 expression and decreased HMG-CoA expression. Further study is needed to determine precisely where and how FPS exerts these effects. FPS offers potential as a therapeutic agent for the treatment of hypercholesterolemia.

## Introduction

Hypercholesterolemia is an important risk factor for atherosclerosis and consequent cardiovascular and cerebrovascular disease. Increased circulating levels of low-density lipoprotein (LDL) underlie the development of atherosclerosis [1,2]. Statins have been shown to effectively lower LDL levels and reduce both mortality and morbidity associated with coronary heart disease by 30% [3]. However, as noted Steinberg and colleagues in a recent review [4], this still leaves a significant percentage of individuals for whom statin therapy will not prevent the occurrence of adverse events. There is an obvious need for more efficacious and alternative treatment options.

Many Chinese herbal medicines contain polysaccharides which can exert a wide range of pharmacological effects, including lipid lowering [5-10]. One such polysaccharide has been extracted from Radix Aconiti Carmichaeli Praeparata in our laboratory, designated as polysaccharide from fuzi (FPS). In previous investigations, we found have that FPS has both immunomodulatory and hypoglycemic effects [11-13]. Specifically we noted that rats treated with FPS exhibited significantly reduced cyclophosphamide-related bone marrow suppression, an increased number of peripheral white blood cells, reduced oxidative damage in the bone marrow and increased gene and protein expression of the antioxidant enzyme Cu-Zn superoxide dismutase [14,15]. Apoptosis in bone marrow cells was also inhibited (as indicated by decreased caspase-9 and -3 levels) via increased expression of the anti-apoptotic Bcl-2 gene and decreased expression of the pro-apoptotic BAX gene. Further to this, after two weeks of treatment with FPS (160 mg/kg), elevations in serum triglyceride, cholesterol and LDL levels induced by high-fat diet feeding were significantly reduced. In this same study, the cholesterol and LDL lowering effects of FPS were similar to that of fluvastatin. The triglyceride lowering effect, however, was increased following FPS compared to fluvastatin treatment. Given its reported lipid-lowering and immunomodulatory effects, FPS may be of potential use as an anti-atherosclerotic agent.

The mechanisms underlying the lipid lowering effects of FPS have not been determined. It is essential that these mechanisms be determined to facilitate further development of this agent as a potential therapeutic. Hence the aim of the present study was to investigate the mechanisms underlying FPS-induced hypolipidemia using a rat model of dietart-induced hypercholesterolemia. Specifically we examined the possibility that altered expression of three key players in hepatic lipid metabolism, the LDL-receptor (LDL-R), 3-hydroxy-3-methyl glutaryl (HMG)-CoA reductase and cytochrome P450 7α-1 (CYP7α-1), may contribute to the lipid lowering effect of FPS.

## Materials and methods

### Animals

Fifty male Wistar rats, aged 8-12 weeks and weighing 100 ± 10 g, provided by the Animal Centre of Sun Yat-Sen University were used. Rats were housed in a temperature controlled environment, with a fixed light/dark cycle. This study was approved by the institutional internal review board.

The rats were randomly divided into one of the five following groups: control; high cholesterol; high cholesterol + 224 mg/kg FPS; high cholesterol + 448 mg/kg FPS; high cholesterol + 869 mg/kg FPS. The dose of FPS for each treatment group was decided according to the results of our preliminary study. Hence there were 10 rats in each experimental group.

### Experimental procedure

Hypercholesterolemia was induced by feeding the rats a diet high in cholesterol for a period of 14 days. The diet contained 2% cholesterol (Probe Biological Technology Ltd. Beijing, China), 0.5% bile salt (AOBO Star Biotechnology Ltd., Beijing, China) and a standard chow mix [16,17]. Rats in the control group were fed a standard laboratory diet, normal in cholesterol.

Rats in the intervention groups were orally gavaged with the appropriate dose of FPS in a volume of 1 ml. Rats in the control and high cholesterol group were gavaged daily with isotonic saline (1 ml).

FPS was extracted in our laboratory as previously described [11].

Body weight, food intake and fecal excretion were measured throughout the study period. Food intake and fecal excretion were determined over 24 h in each case. Fecal samples from the final 3 days of the study were further processed to determine bile acid content.

At the completion of the study, each rat was anaesthetized with intraperitoneal sodium pentobarbital (40 mg/kg; Shanghai Chemical Reagent Company, Beijing, China) and an arterial blood sample was collected. They were then sacrified by cervical dislocation and the liver was then rapidly removed, washed in saline, dried on filter paper and divided into two parts. One part was fixed in 10% formalin and then paraffin-embedded for hematoxylin and eosin staining and immunohistochemistry. The other half was immediately snap-frozen in liquid nitrogen and stored at -80°C for later assessment of HMG-CoA reductase and CYP7α-1 mRNA levels by real-time PCR.

### Real-time PCR [18,19]

Total mRNA was isolated by Trizol reagent according to the procedure of the supplier (Invitrogen, USA). Real-time PCR was performed using SYBR green (Roche, USA) and standard procedures to assess the mRNA expression of HMG-CoA reductase and CYP7α-1 in liver samples obtained from each rat. An Applied Biosystems 7500 real-time PCR machine was used (Applied Biosystems, USA).

The HMG-CoA reductase primer pairs were:

5'-CTT GAC GCT CTG GTG GAA TG-3'

and 5'-AGT TGG AAG CAC GGA CATA-3'

The amplified HMG-CoA fragment was 106 bp, while the reaction parameters were as follows: 10 cycles of 94°C denaturation for 3 min; 94°C denaturation for 60 sec and 55°C annealing for 45 sec.

The CYP7α-1 primer pairs were:

5'-ATG ACC TGC CGG TAC TAG ACA-3'

and 5'-TGA AGT CCT CCT TAG CTG TGC-3'.

The amplified CYP7α-1 fragment was 90 bp, while the reaction parameters were as follows: 30 cycles of 94°C denaturation for 45 sec and 55°C annealing for 30 sec.

### Quantification of bile acid

Fecal samples were dried at 56°C until the weight remained constant. Fifty mg from each sample was added to distilled water (1 ml) and homogenized. Four ml of methanol was then added and the sample was blended in an ultrasonic oven for 1 h, stored at room temperature until the temperature decreased and then centrifuged at 2500 rpm for 10 min. The supernatant (0.5 ml) was removed and mixed with ethanol (1.75 ml), 6% barium hydroxide (0.1 ml) and 10% zinc sulfate (0.05 ml). The sample was then centrifuged at 2500 rpm for a further 10 min and the proteins and pigment in the precipitant removed. A volume of supernatant (0.2 ml) was removed, placed in a glass tube, steam-dried, reconstituted with 0.1 ml of distilled water, 3.9 ml of a chloroform/methanol mixture (2:1) and Folch solution (0.88 ml), mixed thoroughly and left for stratification. The supernatant containing total bile acid (1.8 ml) was withdrawn into a 5 ml tube. The extraction procedure was repeated three times on the remaining solution. Each time, 1 ml of resultant supernatant was removed. Methanol (0.2 ml) was added to the combined volume of supernatant; bringing the total volume to 5 ml. 0.5 ml of this mixture was steam-dried in a glass tube, after which 2 ml of phosphomolybdate acid reagent was added. The solution was then maintained at 80°C in a water bath for 60 minutes, before being cooled in ice water for 5 min to terminate the reaction. Diluent (2 ml) was then added, the solution mixed and absorbance at 690 nm determined using a spectrophotometer.

### Western blotting [20]

Frozen liver was homogenized and centrifuged. Supernatant was collected and protein concentration was determined using a Bio-Rad kit (Bio-Rad Laboratories). Liver extracts (100 ug) were analyzed by Western blot analysis with antibody against rat LDL-R antibody (1:800, Santa Cruze, USA). Blots were reblotted with antibody against GAPDH for a loading control.

### Quantification of serum total cholesterol, triglyceride and high- and low-density lipoprotein concentrations

Total cholesterol, triglyceride, HDL-C and LDL-C serum concentrations were determined using kit methods following the instructions provided (Shanghai Rongsheng Biotechnology Company, Shanghai, China).

### Statistical analyses

Data were compared between groups by one-way analysis of variance. When a significant between groups difference was apparent, multiple comparisons of means were performed using the Bonferroni procedure with type-I error adjustment. Data are presented as means ± standard SD. All statistical assessments were two-sided and evaluated at the 0.05 level of significant difference. Statistical analyses were performed using SPSS 15.0 statistical software (SPSS Inc, Chicago, IL).

## Results

Table 1 summarizes the lipid profiles of rats in each of the five groups. There were significant differences in serum cholesterol and LDL-C levels between the five groups (*P *< 0.05). Serum cholesterol and LDL-C levels in the HC group were significantly higher than those in the control group (*P *< 0.05). Concentrations of serum cholesterol and LDL-C were significantly lower in each of the FPS groups compared to the HC group (*P *< 0.05).

**Table 1:** Summary of lipid profiles in rats fed a high cholesterol diet and treated with different concentrations of FPS.

	Control (n = 10)	HC group (n = 10)	FPS (224 mg/kg) (n = 10)	FPS (448 mg/kg) (n = 10)	FPS (869 mg/kg) (n = 10)	*P*-value^
Cholesterol (mmol/L)	1.33 ± 0.19	2.89 ± 0.48^†^	2.20 ± 0.38^†‡^	1.91 ± 0.46^‡^	1.93 ± 0.37^‡^	<0.001*
Triglyceride (mmol/L)	0.79 ± 0.21	0.85 ± 0.33	0.88 ± 0.38	0.76 ± 0.36	0.87 ± 0.35	0.942
LDL-C (mmol/L)	0.79 ± 0.07	1.97 ± 0.46^†^	1.20 ± 0.15^†‡^	1.13 ± 0.10^‡^	1.27 ± 0.21^†‡^	<0.001*
HDL-C (mmol/L)	1.11 ± 0.17	1.22 ± 0.27	1.19 ± 0.24	1.11 ± 0.26	1.15 ± 0.33	0.830

Data are presented as the mean ± standard deviation.

HC = high cholesterol; FPS = Polysaccharide from Fuzi; LDL = low-density lipoprotein; HDL = high-density lipoprotein.

^*P*-values are based on analysis of variance testing.

* Indicates an overall significant difference (*P *< 0.05).

^† ^Indicates a statistically significant difference between the highlighted treatment group and the control group (*P *< 0.05).

^‡^Indicates a statistically significant difference between the highlighted treatment group and the HC group (*P *< 0.05).

Pair-wise multiple comparisons between groups were determined using Bonferroni's test with α = 0.007 adjustment.

**Figure 1:**
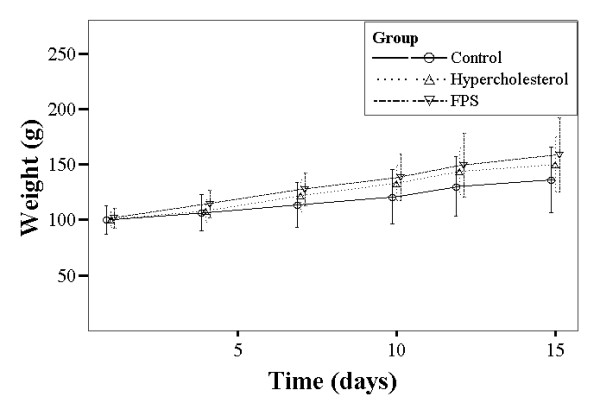
**Summary of body weight changes over the study period for each group of rats**. Control rats were fed a normal diet. Rats in the high cholesterol (HC) group were fed a high cholesterol diet. Rats in the FPS group were fed the high cholesterol diet and were treated with FPS (224 mg/kg) daily. All rats (regardless of group) gained significant weight over the study period (*P *< 0.05).

**Figure 2:**
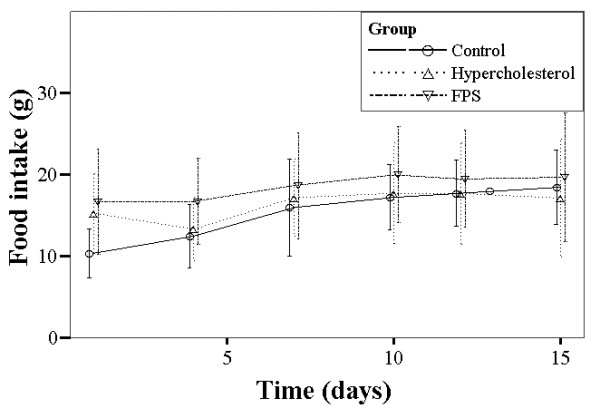
**Summary of 24 h food intake over the study period for each group of rats**. Control rats were fed a normal diet. Rats in the high cholesterol (HC) group were fed a high cholesterol diet. Rats in the FPS group were fed the same high cholesterol diet and were treated with FPS (224 mg/kg) daily.

**Figure 3:**
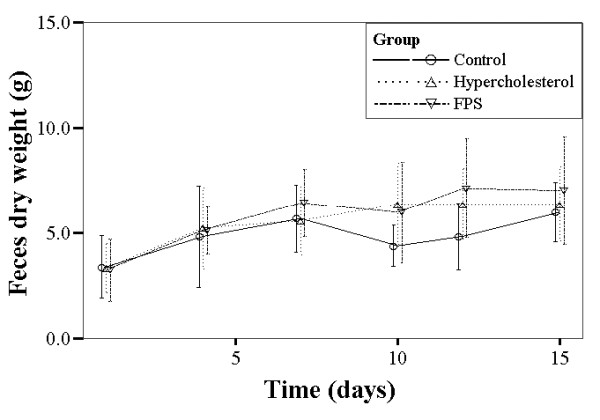
**Summary of 24 h fecal dry weight over the study period for each group of rats**. Control rats were fed a normal diet. Rats in the high cholesterol (HC) group were fed a high cholesterol diet. Rats in the FPS group were fed the same high cholesterol diet and were treated with FPS (224 mg/kg) daily.

Figure 1 summarizes the changes in body weight over the study period for the different groups. For each, body weight increased significantly over the course of the study period (*P *< 0.001), however there were no between group differences. There were no significant within or between group differences in food intake or feces dry weight over the study duration (Figures 2 and 3).

**Figure 4:**
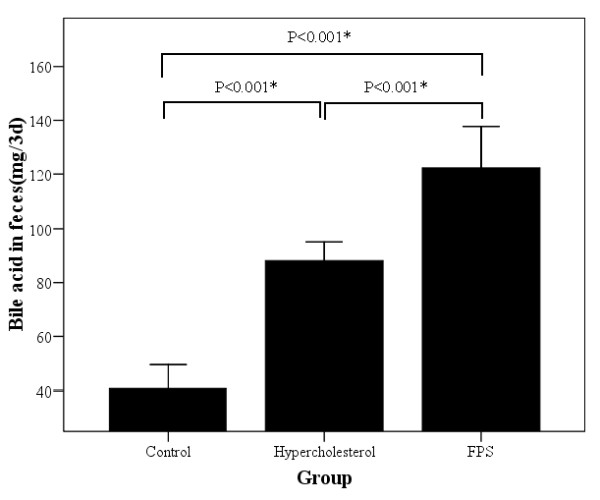
**Summary of fecal bile acid content over the last three days of the study period for each group of rats**. Control rats were fed a normal diet. Rats in the high cholesterol (HC) group were fed a high cholesterol diet. Rats in the FPS group were fed the same high cholesterol diet and were treated with FPS (224 mg/kg) daily. * indicates a statistically significant between groups difference (*P *< 0.05).

There was a significant difference in the content of fecal bile acid among the three 3 groups (Figure 4; *P *< 0.001). Bile acid content was significantly higher in the HC group compared to control group (*P *< 0.001), while levels were significantly higher in the FPS group compared to both other groups (*P *< 0.001).

**Figure 5:**
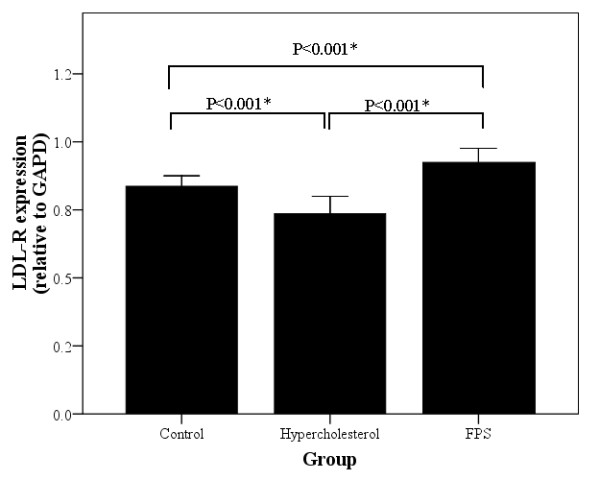
**Summary of hepatic low-density lipoprotein receptor (LDL-R) expression levels for each group of rats as determined at study completion by Western blotting**. Control rats were fed a normal diet. Rats in the high cholesterol (HC) group were fed a high cholesterol diet. Rats in the FPS group were fed the same high cholesterol diet and were treated with FPS (224 mg/kg) daily. * indicates a statistically significant between groups difference (*P *< 0.05).

Figure 5 summarizes liver LDL-R protein expression for each group of rats as determined by Western blotting. Hepatic LDL-R protein expression was significantly decreased in the HC group compared to both other groups (*P *< 0.001), while expression levels were significantly higher in the FPS group compared to the control group (*P *< 0.001).

The hepatic mRNA expression levels of HMG-CoA reductase and CYP7α-1 as determined by real time-PCR are summarized in Table 2. Significant overall differences were apparent for both enzymes (*P *< 0.001). HMG-CoA reductase mRNA expression was significantly higher in the HC group compared to both the control and FPS groups (*P *< 0.05). There was no significant difference between the FPS and control group for this variable. CYP7α-1 mRNA levels were significantly higher in the FPS group compared to both the HC and control groups (*P *< 0.05).

**Table 2:** Summary of hepatic HMG-CoA reductase and CYP7α-1 mRNA expression levels in rats fed a high cholesterol diet and treated with different concentrations of FPS.

mRNA levels	Control (n = 10)	HC group (n = 10)	FPS (n = 10)	P-value^
HMG-CoA reductase	1.29 ± 0.36	4.17 ± 1.83^†^	0.73 ± 0.39^‡^	<0.001*
CYP7α-1	0.78 ± 0.43	0.74 ± 0.49	4.08 ± 1.58^†‡^	<0.001*

Data are presented as the mean ± standard deviation.

HC = high cholesterol; FPS = Polysaccharide from Fuzi;

^*P*-values are based on analysis of variance testing.

* Indicates an overall significant difference (*P *< 0.05).

^† ^Indicates a statistically significant difference between the highlighted treatment group and the control group (*P *< 0.05).

^‡^Indicates a statistically significant difference between the highlighted treatment group and the HC group (*P *< 0.05).

Pair-wise multiple comparisons between groups were determined using Bonferroni's test with α = 0.017 adjustment.

## Discussion

In this study we utilized a rat model of hypocholesterolemia to investigate the mechanisms underlying the lipid-lower effects of FPS, a Chinese herbal medicine extract. In the face of hypocholesterolemia, we found that treatment with FPS not only significantly reduced serum LDL and cholesterol concentrations, but also increased mRNA expression of HMG-CoA reductase and CYP7α-1 in the liver. Further to this, LDL-R expression levels were markedly decreased.

The number and activity of LDL-Rs are important factors that influence the metabolism of LDL [21,22]. This receptor internalizes LDL, thus lowering plasma LDL and cholesterol concentrations [16]. Previous studies have shown that many hypolipidemic drugs affect LDL-R expression [23,24]. Further to this, there are a substantial number of reports indicating that various cholesterol-lowering Chinese herbal medicines also influence LDL-R expression [23]. In the present study, LDL-R immunohistochemical and Western blot analysis was performed on liver tissue obtained from rats fed a high cholesterol diet. We found that the expression of LDL-R was significantly decreased in tissue obtained from rats fed a high cholesterol diet compared to rats fed a normal diet. In contrast, rats fed the high cholesterol diet and treated with FPS exhibited no such decrease in LDL-R expression; rather the levels were significantly increased from those in control rats. Taken together, these findings lead us to suggest that one of the mechanisms through which FPS promotes cholesterol lowering is by inducing LDL-R receptor expression and density, thus enhancing the transfer, conversion and removal of LDL (and cholesterol) from the circulation.

HMG-CoA reductase is the rate-limiting enzyme of the mevalonate pathway, a key pathway of cholesterol synthesis [25]. Indeed inhibition of this enzyme in the liver by statins results in decreased cholesterol synthesis, increased LDL-R synthesis and consequent decreases in circulating levels of LDL and cholesterol [26,27]. In the present study, we found that the significantly increased hepatic HMG-CoA reductase mRNA expression levels in rats fed a high cholesterol diet were normalized by treatment with FPS. Indeed HGM-CoA reductase levels were lower (but not significantly so) in FPS treated rats compared to control rats. Hence it would appear that, similar to statins, the cholesterol lowering effect of FPS may be in part due to inhibition of hepatic HMG-CoA reductase expression. It is unclear precisely how this inhibition may be mediated (direct or upstream). Further studies are needed to determine this.

Our finding that rats fed a high cholesterol diet exhibited increased HMG-CoA reductase expression contrasts with a previous report suggesting that increased cholesterol absorption associated with a high fat diet leads to increased free cholesterol levels in hepatocytes and consequent inhibition of HMG-CoA reductase expression. It is unclear why HMG-CoA mRNA expression was increased in our study.

One of the primary sites of cholesterol metabolism is the liver, where it is converted into bile acid and subsequently excreted in feces. The rate limiting enzyme of this process is CYP7α-1. Indeed increased CYP7α-1 has been demonstrated to hasten the rate of conversion of cholesterol to bile acids in hepatocytes [27,28]. In this study we found that the hepatic mRNA expression levels of CYP7α-1 were significantly elevated in rats treated with FPS as compared to those in the control and high cholesterol groups. This finding indicates that FPS can induce expression of the CYP7α-1 gene. A further test with Western blotting also revealed that the FPS stimulated CYP7α-1 protein expression. In keeping with this finding, the levels of bile acid in fecal samples were markedly elevated in rats treated with FPS, demonstrating that the increased CYP7α-1 expression resulted in heightened cholesterol metabolism. The mechanism(s) through which FPS influences CYP7α-1 expression warrants further investigation.

In this study we have explored several potential mechanisms through which FPS lowers systemic cholesterol levels using a rat model of hypercholesterolemia. We report for the first time that alterations in the hepatic LDL-R, CYP7α-1 and HMG-CoA expression at least in part appear to underlie this effect. Our findings suggest that FPS has the capacity to influence cholesterol handling at multiple points in the metabolic process. Further studies are needed to determine precisely where and how in the various pathways monkshood protein influences expression of the LDL receptor, CYP7α-1 and HMG-CoA reductase. The efficacy of this potential lipid lowering agent also warrants investigation in humans.

## Competing interests

The authors declare that they have no competing interests.

## Authors' contributions

We declare that all the listed authors have participated actively in the study and all meet the requirements of the authorship.

WW designed the study and wrote the protocol. XH managed the literature searches and analyses. XH and WW wrote the first draft of the manuscript. All authors undertook the data collection, and approved the final manuscript.
